# The Comparison of Methods for Bone Reconstruction in the Anterior Wall of the Maxillary Sinus (an Experimental Study)

**DOI:** 10.17691/stm2022.14.1.05

**Published:** 2022-01-28

**Authors:** E.M. Trubushkina, E.M. Boyko, D.V. Stomatov, I.V. Rzhepakovsky, S.I. Piskov, D.S.-A. Yeldashev, A.A. Kutsenko, A.A. Dolgalev

**Affiliations:** Assistant, Department of Otorhinolaryngology and Plastic Surgery with CPE Course; Stavropol State Medical University, 310 Mira St., Stavropol, 355017, Russia;; Lecturer, Essentuki Branch; Stavropol State Medical University, 310 Mira St., Stavropol, 355017, Russia;; Associate Professor, Department of Maxillofacial Surgery, Medical Institute; Penza State University, 40 Krasnaya St., Penza, 440026, Russia;; Leading Researcher; North-Caucasus Federal University, 1 Pushkin St., Stavropol, 355017, Russia; Leading Researcher; North-Caucasus Federal University, 1 Pushkin St., Stavropol, 355017, Russia; PhD Student, Department of General and Pediatric Dentistry; Stavropol State Medical University, 310 Mira St., Stavropol, 355017, Russia;; PhD Student, Department of General and Pediatric Dentistry; Stavropol State Medical University, 310 Mira St., Stavropol, 355017, Russia;; Professor, Department of General and Pediatric Dentistry, Head of the Center for Innovation and Technology Transfer; Stavropol State Medical University, 310 Mira St., Stavropol, 355017, Russia;

**Keywords:** sinus lift, bone tissue, defects in the anterior wall of the maxillary sinus, reparative bone regeneration, materials for regeneration, computed microtomography

## Abstract

**The aim of the study** was to compare various methods used for the bone reconstruction in the anterior wall of the maxillary sinus during sinus lift surgery; in addition, we aimed to study the effect of maxillary sinus membrane perforation on the healing process.

**Materials and Methods:**

The experiments were carried out using the North Caucasian sheep. Maxillary sinus lift surgery was performed on the animals under general anesthesia. The skin and muscle fascia were dissected layer-by-layer providing the optimal conditions for bone preparation; then, three bone windows were made on each side of the head. Two windows were sawn out with a spherical bur, the third window — with a hollow bur and part of the anterior wall was taken out. On one side, the mucous membrane of the maxillary sinus was pulled and perforated; on the other side, the sinus lift was performed with no membrane perforation. On each side, one window was left uncovered, the second was closed with a collagen membrane, and the third was closed with a bone cover. After 30 and 60 days, the sheep were taken out of the experiment in groups of three; samples were collected from the operated areas and examined using computed microtomography and histology.

**Results:**

According to the histological study, the bone repair process developed normally regardless of the surgery technique. The process started with the appearance of granulation tissue and connective tissue cords; in the final stages, cellular differentiation, pronounced osteoblastic activity, and inter-beam formation were seen.

The most active regeneration was observed in the areas where the bone defects were closed with a collagen membrane, and especially in the windows made with no perforation of the maxillary sinus membrane. The microtomographic and histological tests proved that perforation of the mucous membrane during the sinus lift operation impaired bone tissue regeneration.

**Conclusion:**

The obtained results suggest that the most promising way to close a bone defect in the anterior wall of the maxillary sinus is the use of a collagen membrane; therefore, we recommend choosing this approach for sinus lift surgery.

## Introduction

A decrease in the functional load on the jawbone following tooth extraction causes a shift in the remodeling process towards bone resorption. In the lateral parts of the upper jaw, this leads to an increase in the sinus volume due to the decreased height of the edentulous alveolar process. The maxillary sinus pneumatization changes after some of the chewing teeth are removed and it sharply increases after the molar removal. The long post-extraction reparative period, bone resorption, and the sinus pneumatization often result in a reduced height of the maxillary alveolar ridge, making it difficult to place implants and prosthetics in these areas. To increase the volume of bone tissue in the lateral parts of the upper jaw, sinus lift surgery is used [1–8].

Sinus lift or maxillary sinus augmentation, although well documented and fairly predictable, can sometimes become complicated by a sinus membrane perforation non-closure of the lateral foramen, intra- or postoperative bleeding, or augment infection [9–12].

Currently, there are a number of methods for closing the lateral window after sinus lift: e.g., closing the window with collagen membranes, using platelet-rich masses, or bone blocks [1–8]. In the literature, however, there are no convincing data on the advantages or disadvantages of a particular technique.

**The aim of the study** was to compare various methods used for the bone reconstruction in the anterior wall of the maxillary sinus during sinus lift surgery; in addition, we aimed to study the effect of maxillary sinus membrane perforation on the healing process.

## Materials and Methods

The study was carried out with 6 mature sheep of the North Caucasian breed aged 1.5–2 years, weighing 35–40 kg. The sheep were kept in enclosures and fed a regular diet. The experimentation was conducted according to the regulatory technical documents: Order of the Ministry of Health of the USSR of August 12, 1977 No.755 “On measures to further improve the organizational work using experimental animals”, Rules for using experimental animals and Directives 2010/63/EC of the European Parliament and Council of September 22, 2010 on the protection of animals used for scientific purposes.

All manipulations were performed under general anesthesia: sodium thiopental solution was injected intramuscularly at a dose of 50 mg/kg of body weight. The following combination of drugs was used for premedication: Droperidol 0.25% — 0.2 ml/kg; Relanium 0.5% — 0.2 ml/kg; Tramal — 1.0 ml [12].

**Figure 1:**
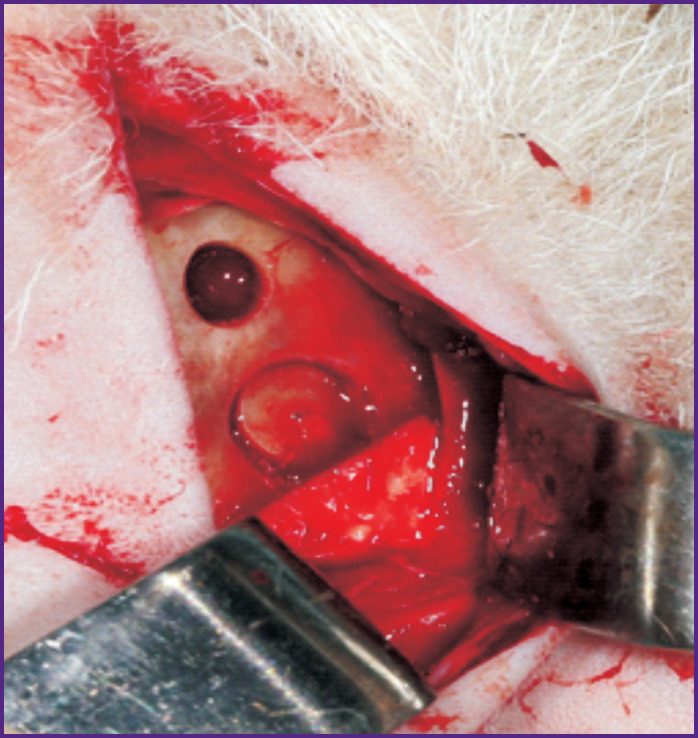
Closure of the bone window with a bone cover (center) and a collagen membrane (right)

After placing the animal on the operating table in the supine position, the hair was shaved off the skin over the maxillary sinus and the surgical field was treated with 5% iodine solution. Then, an external access to the maxillary sinuses was created: the skin and muscle fascia were cut layer-by-layer, providing the optimal conditions for bone preparation; then, three bone windows were made on each side of the head. Two windows (medial and distal) were sawn out with a spherical bur without taking the bone cover out; the third window (in the center) was sawn out with a hollow bur and the bone cover was taken out. On one side of the head, the mucous membrane of the maxillary sinus was pulled and perforated; on the contralateral side, a sinus lift was performed without further perforation. On both sides, the medial bone window remained non-closed, while the distal window was closed with a collagen membrane and the central window was closed with a bone cover. After that, layer-by-layer suturing of the soft tissues was applied according to the standard method (Figure 1).

The choice of sheep for experimental upper jaw surgery was based on: the ease of maintenance and low level of animal aggression, the large surface of available bone tissue in the area of interest, and the high regenerative potential of the osseous donor zones, which made it possible to keep the animal alive after surgery.

In this experiment, the sheep were given soft food 24 h after the operation. During the first 7–10 postoperative days, visual inspection was performed daily. The sutures were removed on day 10. After 30 and 60 days, the sheep were taken out of the experiment in groups of three (overdose of the general anesthetic Zoletil 100 was used). Bone samples for morphological examination were obtained with the help of osteotomes and a circular saw. The samples were fixated in 10% buffered formalin solution as accepted in pathomorphological and histological examinations.

The samples were analyzed using microtomography followed by histological examination. We assessed the bone tissue response to various bioresorbable materials and the parameters of reparative osteogenesis.

### Computed microtomography

To study the skull bone structure and determine the bone mineral density, an X-ray computed microtomograph SkyScan 1176 (Bruker, Belgium) was used.

Scanning of bone samples was carried out in parallel with two phantoms (0.25 and 0.75 g/cm^3^ calcium hydroxyapatite Ca_5_(PO_4_)_3_(OH)) that had a diameter close to the thickness of the sheep skull bones. The scanning parameters were set according to the requirements of the SkyScan 1176, 10.0.0.0 control program (Bruker-microCT, Belgium): X-ray tube voltage — 65 kV, X-ray tube current — 380 μA, filter — 1 mm Al, image pixel size — 17.74 μm, tomograph rotation angle — 180º, rotation increment — 0.3, and frame averaging number — 4.

The digital data were reconstructed using the NRecon 1.7.4.2 program (Bruker-microCT) with the following parameters: smoothing value — 2, ring reduction — 20, beam gain — 36, minimum for converting CS to image was 0.005, and the maximum — 0.05. 3D orientation (x, y, z) and selection of the relevant areas in the reconstructed images were performed using the DataViewer 1.5.6.2 program (Bruker-microCT). Visualization, data analysis, and bone density determination were performed using the CT Analyzer, 1.18.4.0 program (Bruker-microCT).

In accordance with the manufacturer’s recommendations, the program was first calibrated using the phantoms; then, the bone mineral density was determined in various selected parts of the samples. 3D visualization of the scans was performed using the CTvox program, 3.3.0r1403 (Bruker-microCT).

### Histological examination

The obtained samples of bone tissues were preliminarily subjected to non-acidic decalcification: Trilon B was used as a decalcifying agent. After washing under running water for 24 h, tissue samples were dehydrated in isopropyl alcohol, followed by embedding in HISTOMIX medical paraffin (Biovitrum, Russia). Histological sections 5–7 μm-thick were prepared using an MS-2 sledge microtome (ATM-practica, Russia). The prepared sections were stained with hematoxylin and eosin, followed by the standard histopathological analysis.

Tissue micropreparations were viewed under an Axio Imager 2 (A2) (Carl Zeiss Microscopy, Germany) biological microscope at various magnifications; the images were captured using a specialized AxioCam MRc 5 camera and the Zen 2 software (Carl Zeiss Microscopy).

## Results

**Figure 2:**
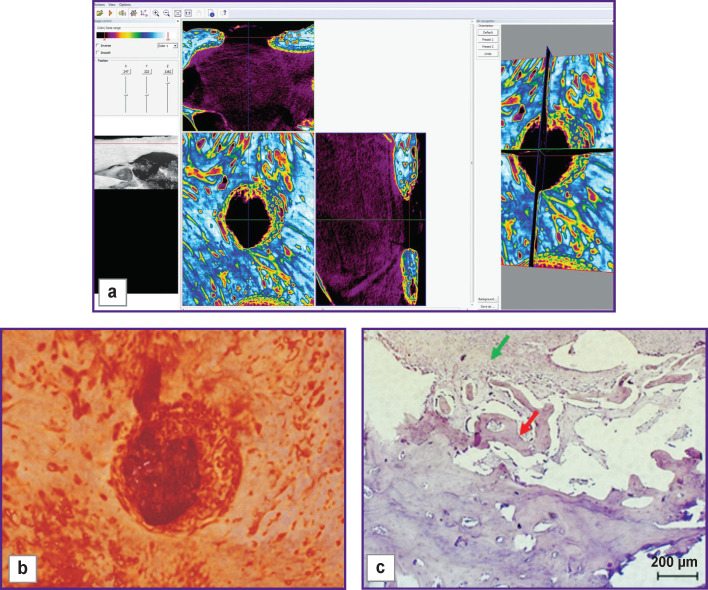
Visualization (a), reconstruction (b), and histological section (c) of a sample from an uncovered bone window with no mucous membrane perforation (day 30 of the experiment)

On day 30 of the experiment, bone samples taken from the windows with no mucous membrane perforation showed the bone defect replaced with various tissues having varying degrees of differentiation. Thus, the defect in the uncovered bone window was completely filled with granulation tissue (Figure 2, *green arrow*) including beams of the newly formed bone tissue and signs of the osteoblastic response (*red arrow*).

**Figure 3:**
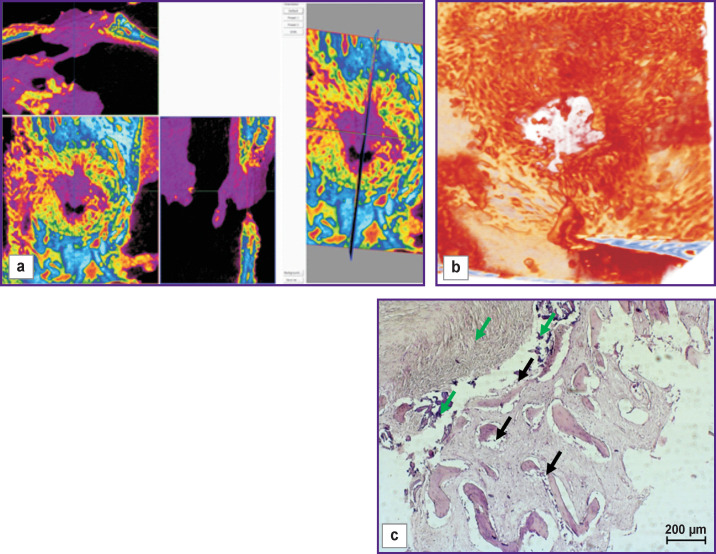
Visualization (a), reconstruction (b), and histological section (c) of a sample from a bone window made with no mucosal perforation and covered with a collagen membrane (day 30 of the experiment)

The bone window closed with a collagen membrane was filled with granulation tissue including bone fragments with signs of resorption (Figure 3, *green arrows*) and signs of the osteoclastic and osteoblastic reactions (*black arrows*). Bone tissue regeneration along the edges was insignificant, and wide areas filled with connective tissue were seen between the newly formed bone beams.

**Figure 4:**
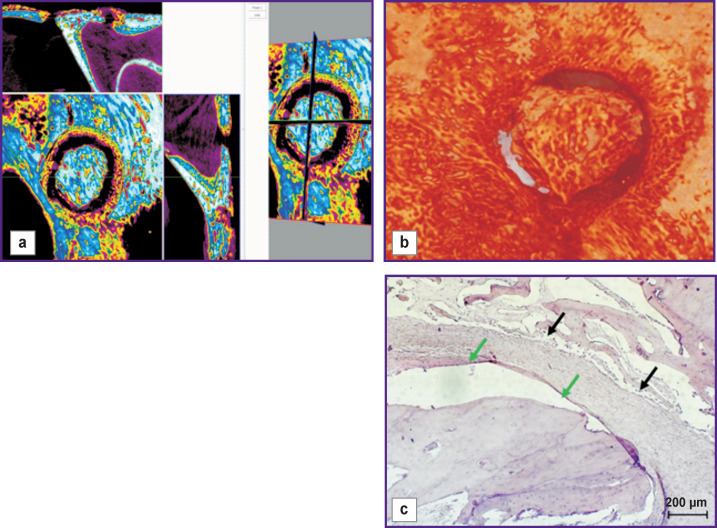
Visualization (a), reconstruction (b), and histological section (c) of a sample from a bone window made with no mucosal perforation and closed with a bone cover (day 30 of the experiment)

The bone window closed with a bone cover was filled with newly formed bone tissue (Figure 4, *green arrows*), in parts, with early signs of bone trabeculae resorption (*black arrow*s). From the top, the defect was replaced with dense compact bone tissue surrounded by cords of coarse fibrous connective tissue adjoined from the outside by trabeculae of newly formed bone tissue with a pronounced osteoblast reaction.

**Figure 5:**
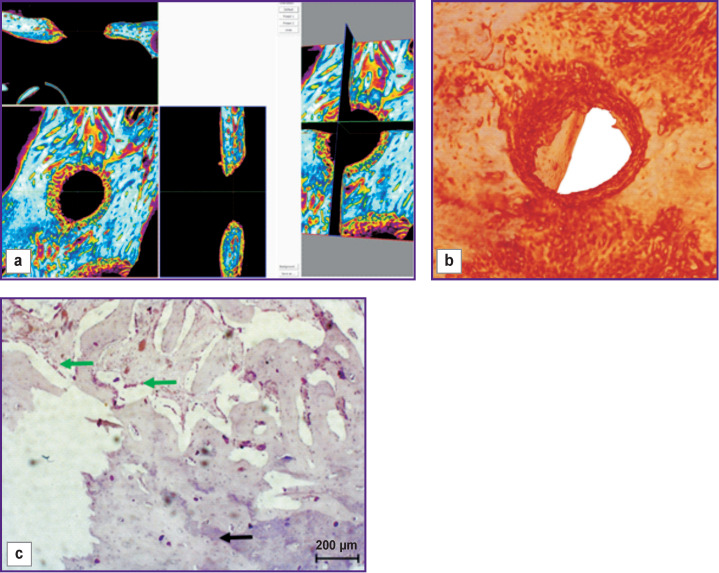
Visualization (a), reconstruction (b), and histological section (c) of a sample from an uncovered bone window made with mucous membrane perforation (day 30 of the experiment)

On day 30 of the experiment, bone samples taken from the windows where the mucous membrane of the maxillary sinus was intentionally perforated, showed a slow defect replacement with various tissues with varying degrees of differentiation. Thus, the non-closed bone window was filled with newly formed bone tissue having signs of an osteoblastic reaction (Figure 5, *green arrows*); in addition, trabeculae with elements of resorption were seen and large areas of dense compact bone tissue were found at the border of the defect (*black arrow*).

**Figure 6:**
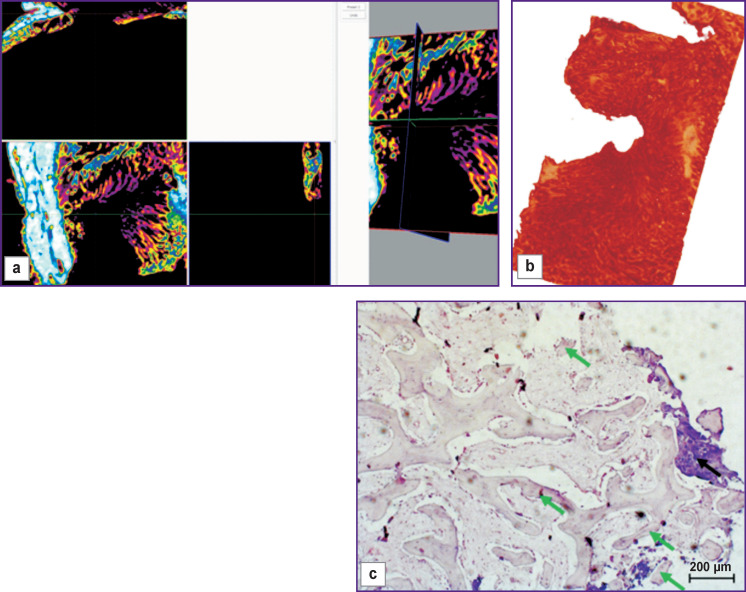
Visualization (a), reconstruction (b), and histological section (c) of a sample from a bone window made with mucosal perforation and covered with a collagen membrane (day 30 of the experiment)

The bone window closed with a collagen membrane was filled with newly formed bone tissue; thinned trabeculae with signs of resorption and osteolysis were detected (Figure 6, *green arrow*s). In some foci, necrosis of the newly formed bone tissue with signs of connective tissue fiber degeneration was also observed (*black arrow*).

**Figure 7:**
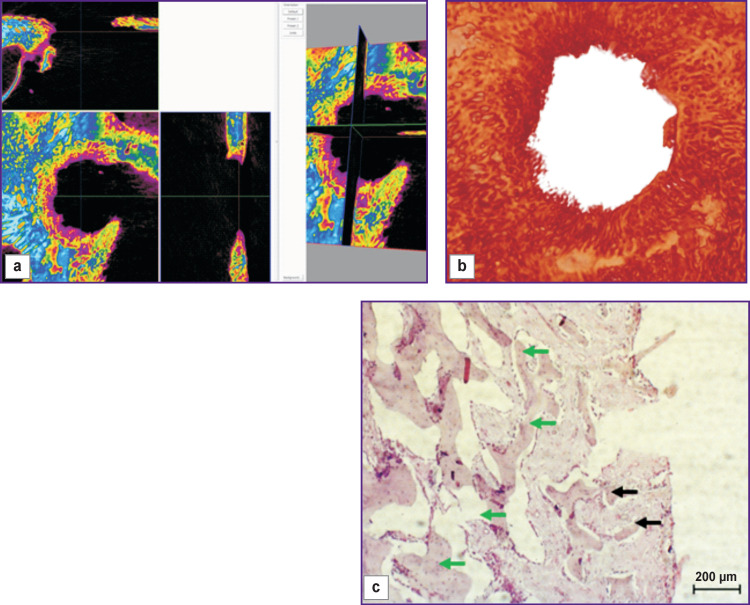
Visualization (a), reconstruction (b), and histological section (c) of a sample from a bone window made with mucosal perforation and closed with a bone cover (day 30 of the experiment)

The bone window closed with the bone cover was filled with newly formed bone tissue having signs of an osteoblastic reaction (Figure 7, *green arrows*); in some places, minor signs of bone trabeculae resorption were detected (*black arrows*).

On day 60 of the experiment, in bone samples taken from the windows made with no membrane perforation, structures of well-formed bone tissue were noted; in addition, early signs of inter-beam connective tissue development could be seen. At the same time, part of the defect was still filled with granulation tissue.

**Figure 8:**
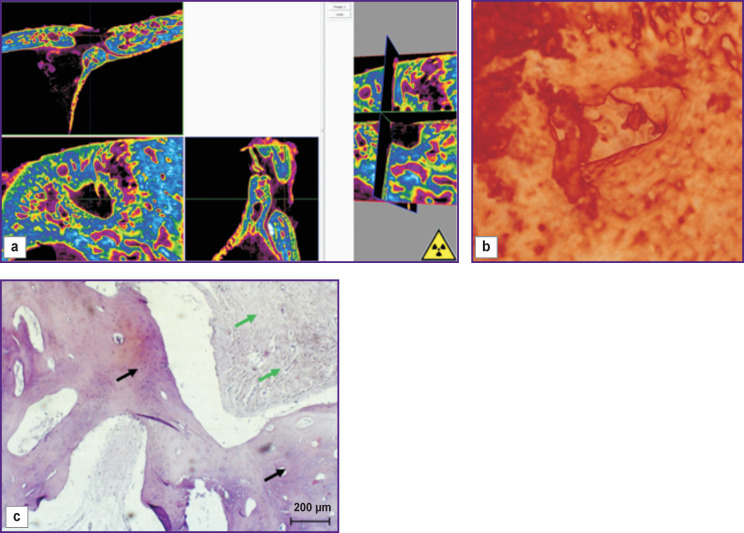
Visualization (a), reconstruction (b), and histological section (c) of a sample from an uncovered bone window made with no mucous membrane perforation (day 60 of the experiment)

Part of the open bone window on day 60 of the experiment was filled with granulation tissue (Figure 8, *green arrows*); in the boundary of the bone defect, structures of well-formed bone tissue with some bone trabeculae in the state of resorption (*black arrows*) were revealed.

**Figure 9:**
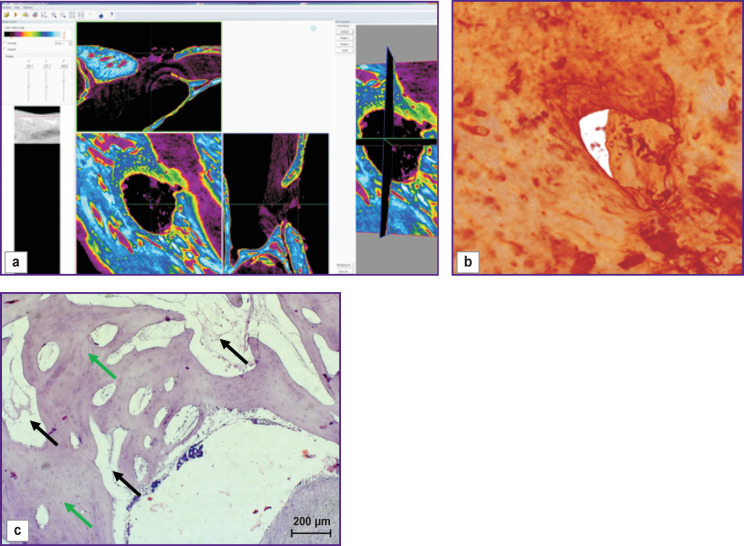
Visualization (a), reconstruction (b), and histological section (c) of a sample from a bone window made with no mucosal perforation and covered with a collagen membrane (day 60 of the experiment)

The bone window closed with a collagen membrane was filled with well-formed bone tissue (Figure 9, *green arrows*), but the inter-beam connective tissue was poorly developed (*black arrows*). Part of the defect was filled with granulation tissue.

**Figure 10:**
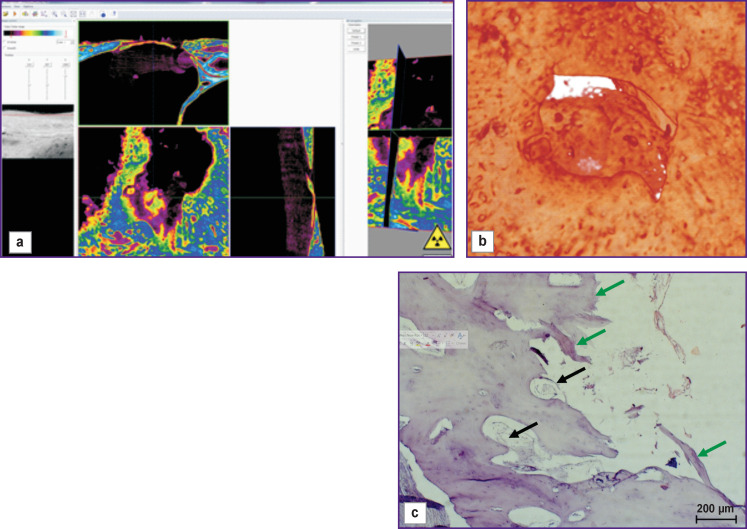
Visualization (a), reconstruction (b), and histological section (c) of a sample from a bone window made with no bone tissue perforation and closed with a bone cover (day 60 of the experiment)

The bone window closed with the bone cover was partially filled with granulation tissue (Figure 10, *green arrows*). At the same time, structures of well-formed bone tissue at the border areas and some trabeculae with primary resorption signs (*black arrows*) were observed.

**Figure 11:**
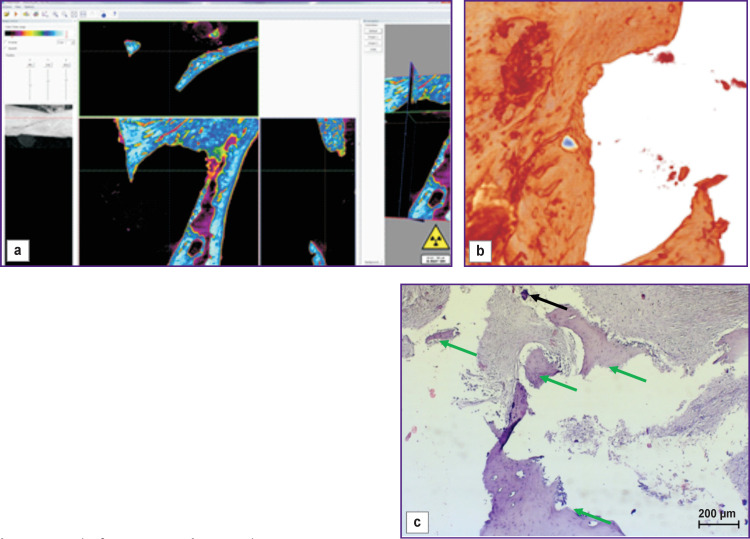
Visualization (a), reconstruction (b), and histological section (c) of a sample from an uncovered bone window made with mucous membrane perforation (day 60 of the experiment)

On day 60 of the experiment, in bone samples taken from non-closed windows where the mucous membrane was perforated, structures of the newly formed bone tissue were found. However, the trabeculae were thinned and showed signs of resorption and osteolysis (Figure 11, *green arrows*). Isolated foci of necrosis were found in the newly formed bone tissue together with dystrophic changes in connective tissue fibers (*black arrow*).

**Figure 12:**
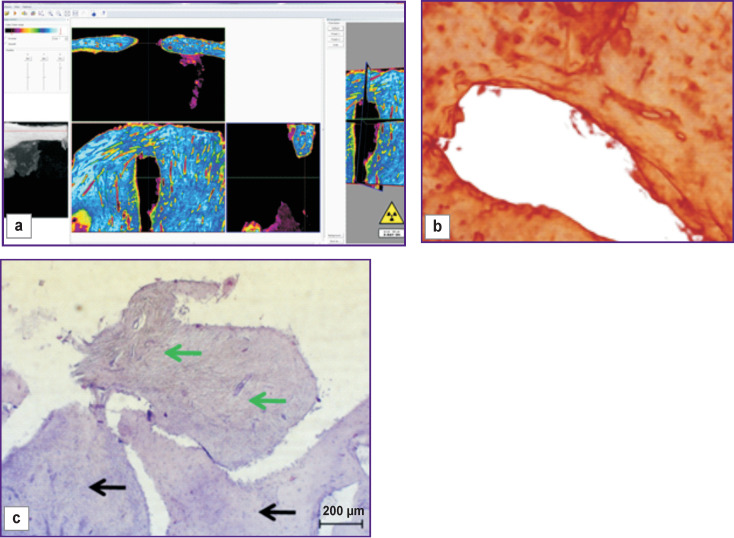
Visualization (a), reconstruction (b), and histological section (c) of a sample from a bone window made with mucosal perforation and covered with a collagen membrane (day 60 of the experiment)

Part of the bone window covered with a collagen membrane was filled with granulation tissue (Figure 12, *green arrows*). Structures of well-formed bone tissue were found at the border of the bone defect (*black arrows*).

**Figure 13:**
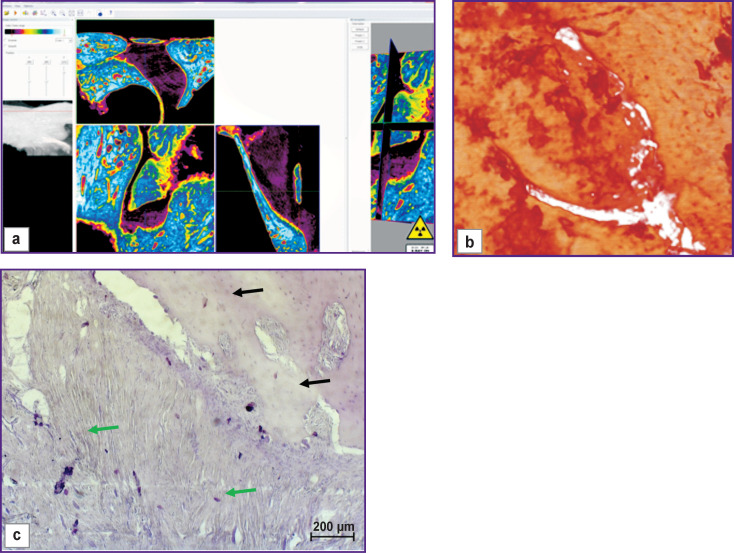
Visualization (a), reconstruction (b), and histological section (c) of a sample from a bone window made with mucosal perforation and closed with a bone cover (day 60 of the experiment)

The bone window, closed with the bone cover, was filled with granulation tissue (Figure 13, *green arrows*), structures of well-formed bone tissue were detected at the bone defect border (*black arrows*).

## Discussion

The obtained results allow us to draw the following conclusions:

1. according to the microtomographic and histological examinations, perforation of the maxillary sinus mucous membrane during sinus lifting impairs bone tissue regeneration;
2. according to the histological examination, in all tested variants of bone defect, bone tissue replacement proceeds according to the normal physiological type; the restoration process begins with granulation tissue containing connective tissue cords and ends by cellular differentiation with a pronounced osteoblastic activity and the formation of an inter-beam system;
3. the most active reparative regeneration occurs in bone defects closed with a collagen membrane, while the non-perforated mucous membrane promotes further regeneration.

## Conclusion

The use of a collagen membrane is the most promising method of closing a bone defect in the anterior wall of the maxillary sinus.

## References

[1] Azarova O.A., Azarova E.A., Kharitonov D.Yu., Podoprigora A.V., Shevchenko L.V. (2019). Modern aspects of application of osteoplastic materials in dental surgery.. Naucnye vedomosti Belgorodskogo gosudarstvennogo universiteta. Seria: Medicina. Farmacia.

[2] Akhmadov I.S. (2020). Patologiya verkhnechelyustnykh pazukh kak faktor riska razvitiya sinusita pri operatsiyakh sinus-lifting..

[3] Vishnyakov V.V., Talalaev V.N., Yalymova D.L (2015). The comparative analysis of the effectiveness of various forms of the surgical treatment of the patients presenting with chronic odontogenic maxillary sinusitis.. Vestnik otorinolaringologii.

[4] Daminov R.O (2010). Maxillary sinus inflammation after operation of dental implantation and sinus lifting.. Stomatologia.

[5] Maksyukov S.Yu., Bojko N.V., Shcheplyakov D.S., Krainyukova L.A., Demidova A.A., Maksyukova E.S. (2016). Diagnostic significance of computed tomography for the detection of odontogenic maxillary sinusitis and the effectiveness of predimplantological alveolar bone ridge augmentation.. Glavnyj vrac Uga Rossii.

[6] Dolgalev A.A., Amkhadov I.S., Atabiev R.M., Tsukaev K.A., Arakelyan N.G., Eldashev D.S (2018). Morphological evaluation of bone tissue under collagen and titanium membranes in experiment.. Medicinskij alfavit.

[7] Zernitskiy A.Yu., Kuz’mina I.V. (2012). Factors affecting the favorable outcome of the sinus lift operation.. Institut stomatologii.

[8] Ivanov S.Yu., Bizyaev A.F., Lomakin M.V., Panin A.M. (1999). Clinical results of the use of various osteoplastic materials in sinus lift.. Novoe v stomatologii.

[9] Dolgalev A.A., Chibisova M.A., Nechaeva N.K., Zubareva A.A., Shavgulidze M.A., Gandylyan K.S., Zelenskiy V.A., Khristoforando D.Yu., Goman M.V., Kutsenko A.P., Arakelyan N.G., Ayrapetyan A.A., Dudarev A.L., Kayzerov E.V. (2019). Konusno-luchevaya komp’yuternaya tomografiya v ambulatornoy stomatologii.

[10] Koroteev A.A. (2007). Eksperimental’noe obosnovanie primeneniya novogo osteoplasticheskogo gelya na osnove kollagena i gidroksiapatita s nekollagenovymi belkami kosti dlya zapolneniya kostnykh defektov chelyustey..

[11] Lazareva A.Yu. (2008). CT diagnosis of polypous rhinosinusitis.. Vestnik otorinolaringologii.

[12] Cricchio G., Sennerby L., Lundgren S (2011). Sinus bone formation and implant survival after sinus membrane elevation and implant placement: a 1- to 6-year follow-up study.. Clin Oral Implants Res.

